# Does in-vehicle automation help individuals with Parkinson’s disease? A preliminary analysis

**DOI:** 10.3389/fneur.2023.1225751

**Published:** 2023-10-13

**Authors:** Wayne C.W. Giang, Haolan Zheng, Beth Gibson, Bhavana Patel, Adolfo Ramirez-Zamora, Sandra Winter, Mary Jeghers, Yuan Li, Sherrilene Classen

**Affiliations:** ^1^Department of Industrial and Systems Engineering, Herbert Wertheim College of Engineering, University of Florida, Gainesville, FL, United States; ^2^Department of Occupational Therapy, College of Public Health and Health Professions, University of Florida, Gainesville, FL, United States; ^3^Department of Neurology, College of Medicine, University of Florida, Gainesville, FL, United States

**Keywords:** Parkinson’s disease, autonomous vehicles, on-road, driving errors, driver rehabilitation, advanced driver assistance systems

## Abstract

**Introduction:**

PD is a progressive neurodegenerative disorder that affects, according to the ICF, body systems (cognitive, visual, and motor), and functions (e.g., decreased executive functions, decreased visual acuity, impaired contrast sensitivity, decreased coordination)—all which impact driving performance, an instrumental activity of daily living in the domain of “Activity” and “Participation” according to the ICF. Although there is strong evidence of impaired driving performance in PD, few studies have explored the real-world benefits of in-vehicle automation technologies, such as in-vehicle information systems (IVIS) and advanced driver assistance systems (ADAS), for drivers with PD. These technologies hold potential to alleviate driving impairments, reduce errors, and improve overall performance, allowing individuals with PD to maintain their mobility and independence more safely and for longer periods. This preliminary study aimed to fill the gap in the literature by examining the impact of IVIS and ADAS on driving safety, as indicated by the number of driving errors made by people with PD in an on-road study.

**Methods:**

Forty-five adults with diagnosed PD drove a 2019 Toyota Camry equipped with IVIS and ADAS features (Toyota Safety Sense 2.0) on a route containing highway and suburban roads. Participants drove half of the route with the IVIS and ADAS systems activated and the other half with the systems deactivated.

**Results:**

The results suggest that systems that assume control of the driving task, such as adaptive cruise control, were most effective in reducing driving errors. Furthermore, individual differences in cognitive abilities, particularly memory, were significantly correlated with the total number of driving errors when the systems were deactivated, but no significant correlations were present when the systems were activated. Physical capability factors, such as rigidity and bradykinesia, were not significantly correlated with driving error.

**Discussion:**

Taken together, these results show that in-vehicle driver automation systems can benefit drivers with PD and diminish the impact of individual differences in driver cognitive ability.

## 1. Introduction

Parkinson’s disease (PD) is an age-related, progressive, neurodegenerative disorder characterized by four cardinal symptoms, i.e., resting tremor, rigidity, bradykinesia, and postural instability (1). PD affects, according to the ICF, body systems (cognitive, visual, and motor), and functions (e.g., decreased executive functions, decreased visual acuity, impaired contrast sensitivity, and decreased coordination)—all which impact driving performance, an instrumental activity of daily living in the domain of “Activity” and “Participation” according to the ICF. Second only to Alzheimer’s disease, PD affects more than 960 adults over the age of 60 per 100,000 population worldwide (2). In the United States, PD affects about 1 million Americans (3). Men are 1.5 times more likely than women to be diagnosed with PD, and the incidence of PD increases with age (3). Worldwide, about 7.5 million persons live with PD, a number predicted to increase to almost 13 million by 2040 (4). In addition, the classic motor difficulties described in PD (i.e., tremor, rigidity, bradykinesia, and postural instability), non-motor symptoms, often not responsive to dopaminergic medications, are a significant source of disability. Non-motor symptoms may include visual deficits, cognitive concerns, depression, emotional and behavioral impairments (e.g., apathy and disinhibition), sleep disorders, and autonomic dysfunction (5, 6). These clinical features of PD affect body functions, but also functional performance, activities of daily living (e.g., eating, dressing), and instrumental activities of daily living (e.g., shopping and driving) (7).

Functional performance of those with PD is impaired by demographic factors, such as age, disease duration, and disease severity (8–11). Other complicating factors include daytime sleepiness (12–14), medication use and side-effects (14), and comorbidities (12). Functional performance deficits resulting from PD, are numerous, and include: deficits in binocular acuity and contrast sensitivity (6, 11, 15); visual scanning and speed of processing (11, 16, 17); cognitive impairments (11, 18, 19); set-shifting and cognitive flexibility (17, 18, 20); and psychomotor speed, including reaction time, slowed walking, and fine motor movements (11, 14, 16, 21). Activity limitations and participation restrictions as evaluated by medical professionals and driver rehabilitation specialists, may negatively impact driving ability (9, 22–25). For example, in a meta-analysis of 50 studies [*N* = 5,410; PD = 1,955, Healthy Controls (HC) = 3,455], the odds of on-the-road test failure were 6.16 (95% confidence interval [CI] 3.79–10.03) times higher; and the odds of simulator crashes 2.63 (95% CI 1.64–4.22) times higher, for people with PD (26). In an evidence based review of 27 studies, Devos et al. (21) found that a combination of visual, cognitive, and motor deficits underlie impaired on-road driving performance in PD. Moreover, participants (mean age 68; mild to moderate stages of PD), were more likely to fail a driving assessment compared to age- and gender-matched controls. Impairment in driving performance may lead to an elevated crash risk (14, 27, 28), even in the early stages of PD (14, 21). However, self-reported real-life crash involvement did not differ between people with PD and HC (odds ratio = 0.84, 95% CI 0.57–1.23, *p* = 0.38) (26), even after controlling for differences in age, sex, driving exposure, and disease severity. Taken together, the literature provides persuasive evidence for substantive driving impairment in PD but is inconclusive about the increase in actual crashes, due to methodological limitations.

In developed regions and communities, driving is a fundamental ADL activity and an important determinant of independence, community participation, and quality of life (29, 30). Of note is that the industry standard for determining fitness to drive, is via a gold standard fitness to drive comprehensive on-road driving evaluation conducted by a certified Driver Rehabilitation Specialist (DRS) (31, 32). However, such services require out-of-pocket payments, and clients may not have access due to the small number (~365) of DRSs in the U.S. Still, the dramatic rise in the number of people living with PD underscores the need for well-supported driving interventions, so drivers with PD can stay on the road longer and safer. A secondary data analysis (33) of driving experiences of people with PD illustrated five themes: how meaningful driving is, the negative quality of life experiences associated with driving cessation, the importance of modifying driving behaviors throughout PD progression, the PD-related factors impacting driving, and vehicle/ community (in)accessibility. These findings have implications for developing technology-based interventions to extend driver fitness, to modify driver behavior, to mitigate the PD related factors impacting driving, and to improve vehicle accessibility.

People with PD may not be able to compensate for the progressive loss of functional visual, cognitive, motor, and other sensory abilities. The current rehabilitation interventions are less than optimal to ensure continued driving (25, 34–36); are only tested on a limited number of drivers with PD (25); and although cognitive (34) and simulator training are feasible (35, 36)—results are not generalizable to ensure that PD drivers can return to driving safely and sustainably. Thus, although the simulator is feasible for driving interventions (35, 36), real-world and large-scale driving intervention studies are lacking.

Although highly autonomous vehicles have the potential to enhance people with PD’s mobility and greatly prevent crashes, they may not be available for use for some decades (37, 38). However, in-vehicle automation technologies, now available in most vehicle makes and models, are becoming ubiquitous in everyday use. Exciting opportunities exist to overcome impaired driving performance given the benefits of automated vehicle technologies. Specifically, the Society of Automotive Engineer (SAE) International (39) indicates six levels (Level 0–5) of automation. Level 0 (warnings and momentary assistance to the driver), Level 1 (steering or brake/ acceleration support to the driver), and Level 2 (steering and brake/acceleration support to the driver), may enable the PD driver to resume control of his/her fitness to drive abilities. Likewise, SAE level 3 (driver must be ready to take control of the vehicle at any given time), may yield more risks than benefits for the driver. Level 4 (automated system can perform all driving functions under certain conditions) and Level 5 (automated system can perform all driving functions, under all conditions), may yield multiple benefits related to transportation equity for people with Parkinson’s but is still a decade or more away.

These in-vehicle automation technologies, including in-vehicle information systems (IVIS, usually SAE level 0) and advanced driver assistance systems (ADAS, SAE level 1 or 2), hold plausible opportunities for people with PD. In-vehicle information systems, e.g., lane departure warning, provide warnings to drivers about surrounding road conditions but do not assume tactical or operational functions of the driving task (40)—an action potentially very helpful for a person with PD with compromised cognitive resources. ADAS are integrated systems that interact with drivers to assist with tactical and operational vehicle control in high-risk situations (40). For example, adaptive cruise control helps maintain vehicle time headway speed and lane position—and as such prevent driving errors and mitigate the potential crash risk, as speed and lane position are compromised in people with PD. (21, 22) Researchers conducted a scoping review, to examine the effect of IVIS and ADAS on the driving performance of older adults (65 years of age and older) in driving simulators and on the road (42). Twenty-four studies addressed 15 unique IVIS features and findings indicated improved safety of the driving task (e.g., faster responses) if cognitive workload was not compromised or the driver was not over-reliant on the feature (42). Five studies addressed five unique ADAS features, and findings indicate improved driving task safety and comfort, including speed control, lane maintenance, braking, and decreased driving stress. Only one study (41) involved people with PD and demonstrated improved speed control with the use of a Heads-Up Display system (IVIS). Therefore, drivers with PD may benefit from in-vehicle technologies to mitigate functional performance deficits, decrease driving errors, and enhance their driving performance and ability to stay on the road longer and safer, but no such studies have yet been conducted.

The overarching objective of the study is to quantify the effect of IVIS and ADAS on driving safety as indexed by the number of driving errors made by people with PD as indicators of improved driving performance in an on-road test vehicle. *Hypotheses*: Drivers with PD will demonstrate fewer total number of driving errors (primary outcome) and fewer speeding, lane exceedances and signaling errors, when driving with autonomous in-vehicle technology in an on-road test vehicle. *Rationale*: Our work (42) and that of others (21, 41, 43–45) have shown that drivers with PD make more driving errors, in the simulator (15) and on-road (6, 17, 22, 23, 25), when compared to healthy controls. This inquiry is addressing the above objective via a preliminary *descriptive analysis of on-road errors* detected by the assessment data of the certified driver rehabilitation specialist (DRS). Specifically, in this inquiry, we examined an initial sample of 45 adults with diagnosed PD and compared the number of errors made during an on-road driving course. Errors were identified during the drive by a certified DRS and compared across different segments of the drive (highway and suburban) under conditions where the driving assistance system was activated or deactivated. In addition, we examined the relationship between individual difference factors, such as demographics and cognitive and physical ability, and the number of driving errors made.

## 2. Materials and methods

### 2.1. Participants

Forty-five (*M_age_* = 67.53 years, *SD* = 9.15 years) completed the study in this preliminary analysis. The inclusion criteria were: (1) a diagnosis by a neurologist/movement disorder specialist with clinically probable PD by Movement Disorders Society (MDS) criteria; (2) a mild or moderate disease severity, based on the MDS-Unified Parkinson’s Disease Rating Scale for motor symptoms; (3) between 35 and 85 years of age; (4) currently driving with a valid driver’s license and meets the Florida state requirement for visual acuity of at least 20/50 in one eye, if one eye is blind or 20/200 or worse the other eye must be 20/40 or better (20/40 in at least one eye), minimum acceptable field of vision is 130 degrees; (5) lives independently in the community; (6) are proficient in reading/speaking English; (7) and a Montreal Cognitive Assessment (MoCA) score of 20 or higher (46, 47). Participants were excluded if they had: (1) concurrent neurological conditions (e.g., stroke, uncontrolled seizures; dementia); (2) severe psychiatric (e.g., psychoses or severe anxiety) or physical conditions (e.g., missing limbs) that would preclude full participation; (3) use of psychotropic medications that may negatively affect mental or physical functioning, due to direct or side effects; (4) severe, unpredictable motor fluctuations; and (5) severe sleep difficulties.

Participants were recruited through referrals from two different sources: (1) direct referrals from our team of neurologists affiliated with the UF Fixel Institute for Neurological Disease, and (2) referrals from outside physicians recruited through interactions with local PD patient support groups, mailing lists, and outreach events. All outside referrals were verified by our team of UF neurologists. Each participant was then screened using the inclusion and exclusion criteria listed above. If participants met the telephone inclusion criteria, they were enrolled in the study and scheduled for the experiment. On the day of the experiment, subjects were evaluated using the MoCA, and if they scored below 20, they were excluded from the study (46, 47). Furthermore, all participants were in the *ON* state of medications for at least 1 h.

This project was approved by the UF Institutional Review Board (IRB#: 202002321) and each participant consented to participate in this study by signing an IRB approved informed consent form. Drivers with PD was reimbursed $30.00 for completion of the study.

### 2.2. Design

**Figure 1:**
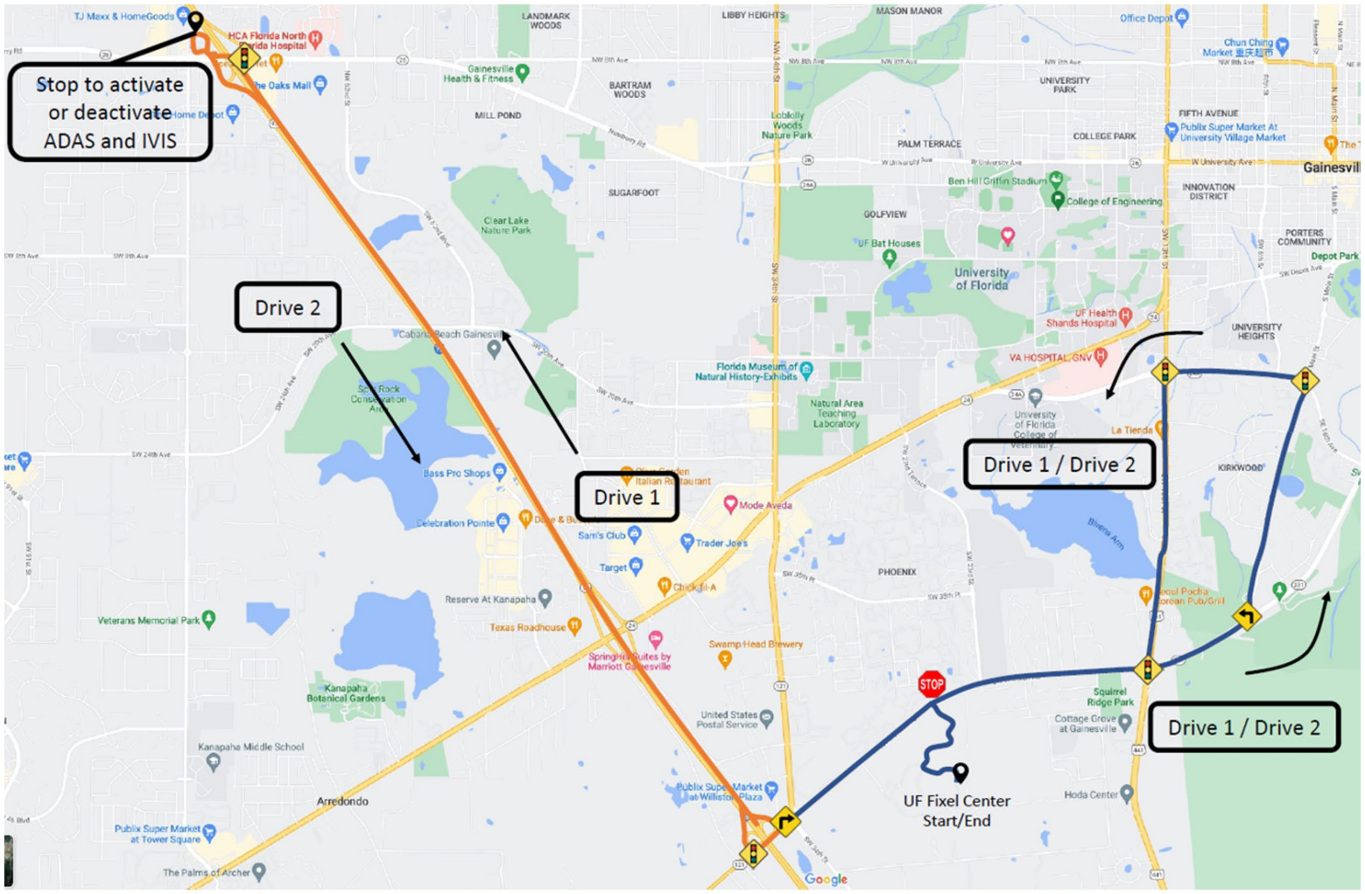
Map of the driving route. Orange denotes highway driving, while blue denotes suburban driving.

The experiment used a within-subjects design where the main experimental variable was the status of the automation systems (activated vs. deactivated). The order of presentation of the system on vs. system off conditions were randomly allocated to participants during recruitment. The IVIS and ADAS systems used was the Toyota Safety Sense 2.0 system found in the 2019 Toyota Camry. This included adaptive cruise control (ACC), lane keeping assist (LKA), lane departure warnings (LDW), and blind spot monitoring (BSM). These systems represent common safety and driver assistance systems found in modern consumer vehicles. Participants drove on both local suburban two-lane and four-lane roadways and a divided highway (Figure 1). The main dependent variables were the number of driving errors made under each experimental condition (system activated vs. system de-activated). Five different types of errors were recorded: overspeeding errors (5 mph over the posted speed limit), underspeeding errors (5 mph under the posted speed limit or below the flow of traffic), encroachment errors (lane departures into oncoming traffic), wide errors (lane departures away from oncoming traffic), and signaling errors (failure to activate or deactivate the turn signal as appropriate).

### 2.3. Experimental apparatus

**Figure 2:**
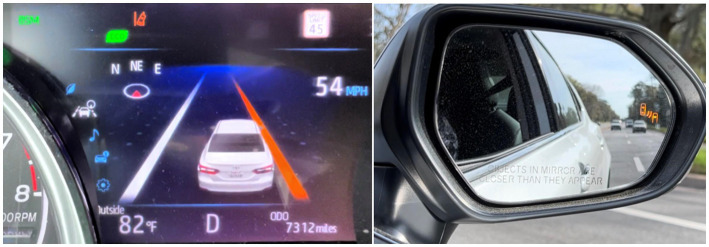
In-vehicle interface showing a lane departure warning (left) and side mirror showing an active blind spot monitor (right).

A Toyota Camry XLE 2019 model (Toyota Motor Cooperation; Tokyo, Japan) featuring the Toyota Safety Sense 2.0 IVIS and ADAS system was used in this study. The IVIS systems include Lane Departure Warnings (LDW), which alerts the driver when the vehicle drifts out of the lane through visual and auditory alerts (Figure 2, left), and Blind Spot Monitoring (BSM), which monitor for vehicles in the car’s blind spots and provides a visual indicator on the side mirrors when a vehicle is present (Figure 2, right).The ADAS systems includes the ACC, which provides cruise control and maintenance of safe vehicle headway distances at speeds above 32 mph, and LKA, which provides gentle steering assistance to bring the vehicle back into the lane when it begins to drift out of the lane. An auxiliary passenger side brake was installed to allow for the DRS to assume control when needed.

### 2.4. Experimental procedures

After providing informed consent, the participants underwent a battery of clinical assessments to ensure they met the inclusion criteria outlined above [MoCA, Snellen chart (48, 49), Optec 2500 Visual Analyzer for field of view (50)]. Eligible participants then completed a demographic questionnaire, and the Movement Disorder Society-Sponsored Revision of the Unified Parkinson’s Disease Rating Scale, Part 3 (MDS-UPDRS Part 3) (51, 52), and the Modified Hoehn and Yahr (MH&Y) disease severity scale (53) was completed by a movement disorders neurologist. Prior to the start of the on-road experiment, the DRS oriented participants to the test vehicle, check for participant proficiency in managing the vehicle’s controls, such as the steering, brake, and gas pedals, and provided a verbal explanation of the ADAS and IVIS driver assistance systems. Each participant also completed a standardized 7-min acclimation drive, to ensure comfort and competency with the vehicle and its controls, within a parking lot before starting the experimental drives.

To control for order and learning effects, two congruent drives were used, which formed one continuous route (Figure 1). Half the participants started with the driver assistance systems activated for the first half of the drive and the systems de-activated during the second half of the drives; the other participants had the order of the system activations reversed. The experimental drive began in the parking lot, leading to a suburban area, and a divided highway section. At the half-way point of the route, after the highway section, participants pulled over in a parking lot and the driver assistance systems were activated or de-activated, depending on the order of the experimental condition. During the system activated condition, the LDW, LKA, and BSM were active in both the suburban and highway road segments, while the ACC was only active during the highway drive. To control for participant skills in operating the ACC, the ACC was set and activated by the DRS once the car had reached an appropriate speed on the highway. The road course included 27 controlled intersections (i.e., traffic signs or signals), 88 uncontrolled intersections, 10 left turns, and five right turns. The speed limits varied between 10 and 45 mph on two-lane and four-lane roadways and was 70 mph on the divided highway. The road course was 26.5 miles in driving distance and took approximately 45 min to drive, depending on the traffic flow. All drives occurred outside of peak traffic hours (i.e., between 9.00 am and 4.00 pm and in the absence of heavy rain or fog).

During the drive, the DRS provided instructions and navigation guidance to the drivers, monitored the drive for safety, and setup the IVIS and ADAS systems when needed. The DRS was also responsible for recording driving errors that occurred during the experiment. The DRS logged the number of errors for each road segment in a standardized paper data recording form that can be seen in the Supplementary material. Two DRSs took part in the data collection and both were provided training to standardize their ratings.

### 2.5. Data analysis and management

Data are displayed descriptively with counts for nominal and ordinal data; and sample means and standard deviations for numerical data. Additionally, boxplots are presented to show the quantiles, median, mean, and individual observations. Error data, aggregated and within each error category, was broken down by system condition (activated vs. deactivated) and driving environment. Driving environment was divided between suburban and highway segments due to the different ADAS and IVIS systems that were active in each road segment. Due to the lower speeds in the suburban section, ACC was not active in the automation activated condition. However, ACC was active in the highway portion of the automation activated drive.

Spearman correlations were used to understand the effects of individual differences within the sample (e.g., age, gender, disease severity, and the clinical tests results) on the number of driving errors when the IVIS and ADAS systems were activated and deactivated. Finally, for each individual difference factor that was significantly correlated with the number of errors, descriptive data and boxplots are provided to show how errors differed across different levels of severity of the factor.

During the experiments, the total scores of both the MoCA and the MDS-UPDRS Part 3 were obtained and computed. The present manuscript provides detailed information on the subtest scores of MoCA and MDS-UPDRS Part 3. Specifically, the MoCA subtests include Visuospatial/Executive and Memory Index Score (MIS), whereas the UPDRS Part 3 subtests include Rigidity, Bradykinesia, and Tremor. The MoCA-MIS, which has a total score of 15 points, was calculated based on the participant’s ability to recall the five words from the memory test after 5 min. Three points were awarded if the participant was able to recall one word without any cues, two points if a category cue was needed, and one point if a multi-choice cue was required. The MDS-UPDRS 3-Rigidity was determined by the summation of subcategories 3.3, while the MDS-UPDRS 3-Bradykinesia was computed by adding up subcategories 3.4–3.8. The MDS-UPDRS 3-Tremor score was calculated by summing up subcategories 3.15–3.18.

Data were captured using the university’s Research Electronic Data Capture (REDCap) system. REDCap is a secure, Health Insurance Portability and Accountability Act compliant web-based application, designed to support data capture for research studies. REDCap provides: (1) an intuitive interface for validated data entry; (2) audit trails for tracking data manipulation and export procedures; (3) automated export procedures for seamless data downloads to common statistical packages such as Microsoft Excel, SAS, Stata, R, or SPSS; and (4) procedures for importing data from external sources. To access the system, all participants were assigned a unique participant ID number and all data from the paper questionnaires and on-road error form were entered and stored by the corresponding participant ID number. Data containing HIPAA identifiers were only accessible to the project team members who have been approved by the IRB.

## 3. Results

### 3.1. Participant demographics

Table 1 shows our participant demographics and the measures of cognitive and physical capabilities in the sample. Our participants were mostly male, and almost all scored mild on the MHYS PD severity scale.

**Table 1:** Participant demographics.

	Number of participants
Gender	*Male:* 31, *Female:* 14
PD Severity (MHYS)^1^	*Mild:* 43, *Moderate:* 2
MoCA^2^	*Normal (≥ 26):* 26, *Impaired (< 26):* 19
MoCA—Executive/Visuospatial^3^	*(VS = 5/5):* 22, *(VS = 4/5)*: 13, *(VS ≤ 3):* 10
MoCA—MIS^4^	*(MIS = 15/15):* 11, *(MIS = 14/15):* 10, *(MIS ≤ 13)*: 24
ON—MDS-UPDRS—Rigidity^5^	*(Rigidity ≤ 2):* 17, *(Rigidity 2–4):* 17*, (Rigidity > 4):* 11
ON—MDS-UPDRS—Bradykinesia^6^	*(Bradykinesia ≤ 5):* 11, *(Bradykinesia 6–10):* 14, *(Bradykinesia > 10)*: 20
ON—MDS-UPDRS—Tremor^7^	*(Tremor ≤ 5):* 36, *(Tremor 6–10):* 5, *(Tremor > 10)*: 4
ON—MDS-UPDRS—Part III Movement Exam^8^	*(ME ≤ 20):* 21, *(ME 21–40):* 21, *(ME > 40)*: 3

^1^MHYS: Modified Hoehn and Yahr Scale.^2^MoCA: Montreal Cognitive Assessment, lower scores indicate greater cognitive impairment.^3^Lower scores indicate greater impairment.^4^Memory Index Score, lower scores indicate greater impairment.^5–8^Higher scores indicate greater impairment, measures were taken with medication in an ON state.

### 3.2. Total number of driving errors

The total errors, summed across the five error categories (overspeeding, underspeeding, wide, encroach, and signal), were compared across the system’s activated and de-activated conditions. The errors were further broken down into the highway portion of the drive, where all automation systems were active (ACC, LDW, LSA, and BSM) in the automation activated condition, and the suburban portion of the drive, where the ACC system was not activated but the remaining systems (LDW, LSA, and BSM) were present.

**Figure 3:**
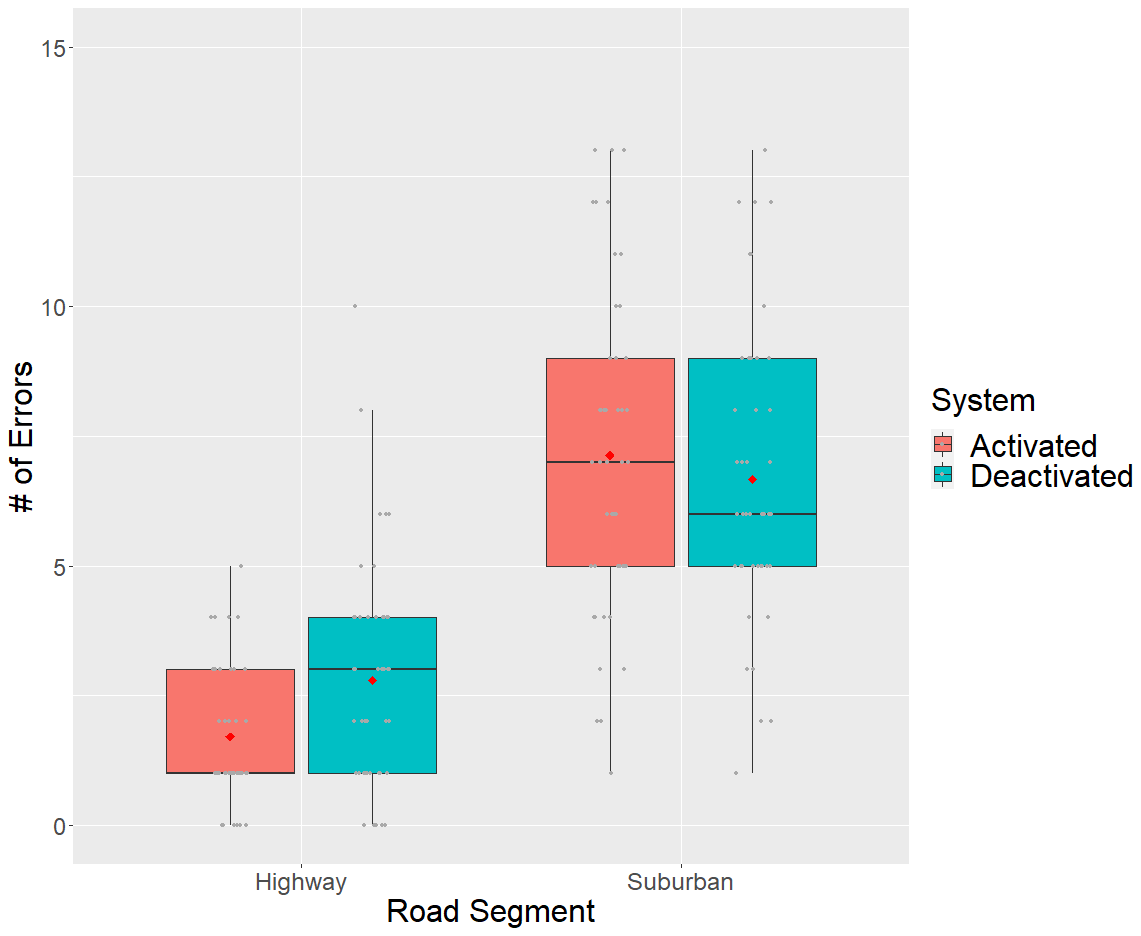
Boxplot of the total errors per participant across driver assistance system status and road segment conditions. The red dot denotes sample means, grey dots denote individual observations.

The descriptive results showed that (Figure 3), for the highway segments, there appeared to be fewer errors made when the ADAS and IVIS systems were activated (M = 1.7, SD = 1.3) than when they were deactivated (M = 2.8, SD = 2.2). As expected, for the suburban section, and due to the longer road segments, participants had more driving errors during the suburban than the highway portion of the drive. Surprisingly, within the suburban portion of the drive, there appeared to be a similar number of errors made when the ADAS and IVIS systems were activated (M = 7.2, SD = 3.2) and when they were deactivated (M = 6.8, SD = 2.9). Overall, these results suggest that the driver assistance systems may be most useful for highway driving.

### 3.3. Types of errors

To better understand the relationship between the IVIS and ADAS and the number of driving errors made by participants, errors were examined across the five error categories (Table 2).

**Table 2:** Number of errors across different types of driving errors (means and standard deviations).

Driving Error	Highway		Suburban	
	System activated	System deactivated	System activated	System deactivated
Overspeeding	0.02 (0.15)	0.09 (0.29)	1.98 (1.84)	1.80 (1.47)
Underspeeding	0.24 (0.53)	0.98 (1.01)	1.87 (2.12)	2.00 (2.27)
Encroach	0.58 (0.75)	0.42 (0.69)	1.11 (1.15)	1.16 (1.22)
Wide	0.76 (1.03)	1.13 (1.63)	2.04 (1.80)	1.58 (1.50)
Signaling	0.11 (0.32)	0.18 (0.49)	0.13 (0.34)	0.18 (0.53)

Overall, there were fewer signaling errors during the drive than the other error types, and little variation occurred regardless of the activation status of the system or the road segment conditions. Both types of speeding errors (i.e., overspeeding and underspeeding), and both types of lane departure errors (encroach errors, wide errors) had more occurrences during the suburban than highway drive segments. Furthermore, there were very few overspeeding errors that occurred in the highway road segments.

### 3.4. Effect of individual differences on error counts

Demographic and individual difference factors, such as age, cognitive, and motor ability, may influence driving errors (14, 17, 19, 21). Spearman correlations were conducted between these individual difference factors and the total number of driving errors that occurred when the IVIS and ADAS systems were activated and when the systems were deactivated (Table 3). Significant correlations would indicate that the corresponding individual difference factor influences the likelihood of driving errors.

**Table 3:** Spearman correlations between individual difference factors and the total number of driving errors with the system activated and deactivated.

Individual difference factors	System activated	System deactivated
Age	0.28 (*p* = 0.06)	0.31^*^ (*p* = 0.04)
MoCA^1^ (total score)	−0.16 (*p* = 0.30)	−0.45^*^ (*p* = 0.002)
MoCA—Executive/Visuospatial	−0.03 (*p* = 0.85)	−0.25 (*p* = 0.10)
MoCA—MIS^2^	−0.26 (*p* = 0.09)	−0.38^*^ (*p* = 0.01)
MDS-UPDRS^3^—Rigidity	−0.10 (*p* = 0.50)	−0.18 (*p* = 0.23)
MDS-UPDRS—Bradykinesia	−0.16 (*p* = 0.31)	−0.21 (*p* = 0.17)

^1^MoCA, Montreal cognitive assessment.^2^MIS, Memory index score.^3^MDS-UPDRS, Movement disorder society unified parkinson’s disease rating scale.^*^denotes significant correlations, *p* < 0.05.Bolded values indicate significant correlations, *p* < 0.05.

As can be seen in Table 3, none of the individual difference factors resulted in significant correlations to the total number of errors when the driver assistance systems were activated. However, when the system was deactivated, increases in Age were correlated with the total number of errors. Furthermore, both the total MoCA score and the MIS component of the MoCA, which measure memory and delayed recall, were negatively correlated with the total number of driving errors. The motor capability factors, measured through the UPDRS rigidity and bradykinesia scores were not significantly correlated with errors.

Since both the MoCA and the MIS subscale within the MoCA appeared to influence the number of total driving errors, further exploratory data analysis was conducted on these variables. MoCA scores were used to divide the participant sample into a lower score group (MoCA <26, *n* = 19) and a higher score group (MoCA ≥26, *n* = 26). MoCA scores of 26 or greater have typically been used as a cut-off for normal function, with scores between 18 and 25 indicating possible mild cognitive impairment (46, 47). The lowest MoCA score within our sample was 20. For MIS, the sample was divided into three groups: perfect scores of 15 (*n* = 11), near perfect scores of 14 (*n* = 10), and scores below 14 (*n* = 24). The MoCA-MIS was calculated by adding the number of words remembered in free delayed recall, category-cued recall, and multiple choice-cue recall multiplied by 3, 2, and 1, respectively, with a score ranging from 0 to 15 (53). A lower MIS score may indicate that an individual was experiencing difficulties with memory (54), which can impact their daily functioning and driving performance.

**Figure 4:**
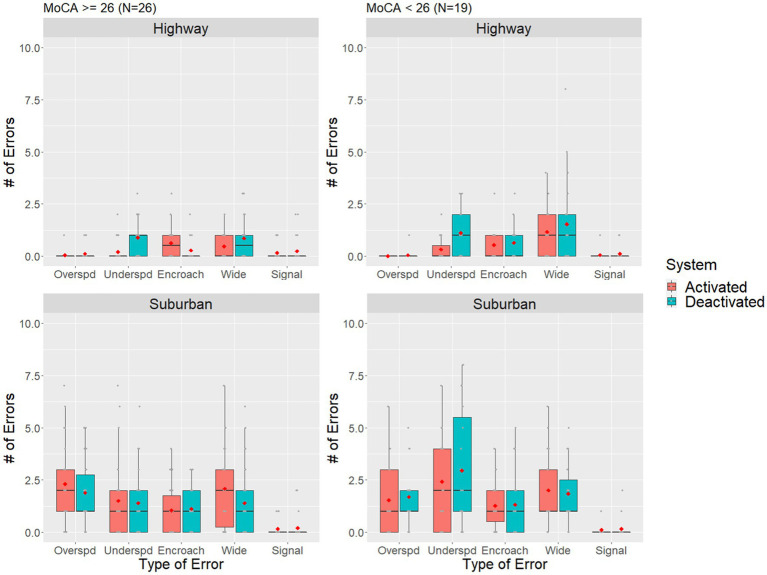
Boxplot of the total number of driving errors for individuals who had higher and lower MoCA score compared across driver assistance system status and road segment conditions. The red dot denotes sample means, grey dots denote individual observations.

Figure 4 suggests that individuals with lower MoCA scores tended to make more driving errors in general than those with higher scores in both highway and suburban driving. Furthermore, and similar to our earlier analysis, the strongest benefits of the driver assistance systems appear to be for underspeeding errors in the highway segment. For most other error types across both highway and suburban driving, the differences between the system activated and system deactivated conditions were not as pronounced. These results suggest that the ACC was beneficial for individuals with lower MoCA scores by helping reduce underspeeding errors, while the alert based IVIS systems (e.g., LDW) had less of an effect on the number of errors experienced.

**Figure 5:**
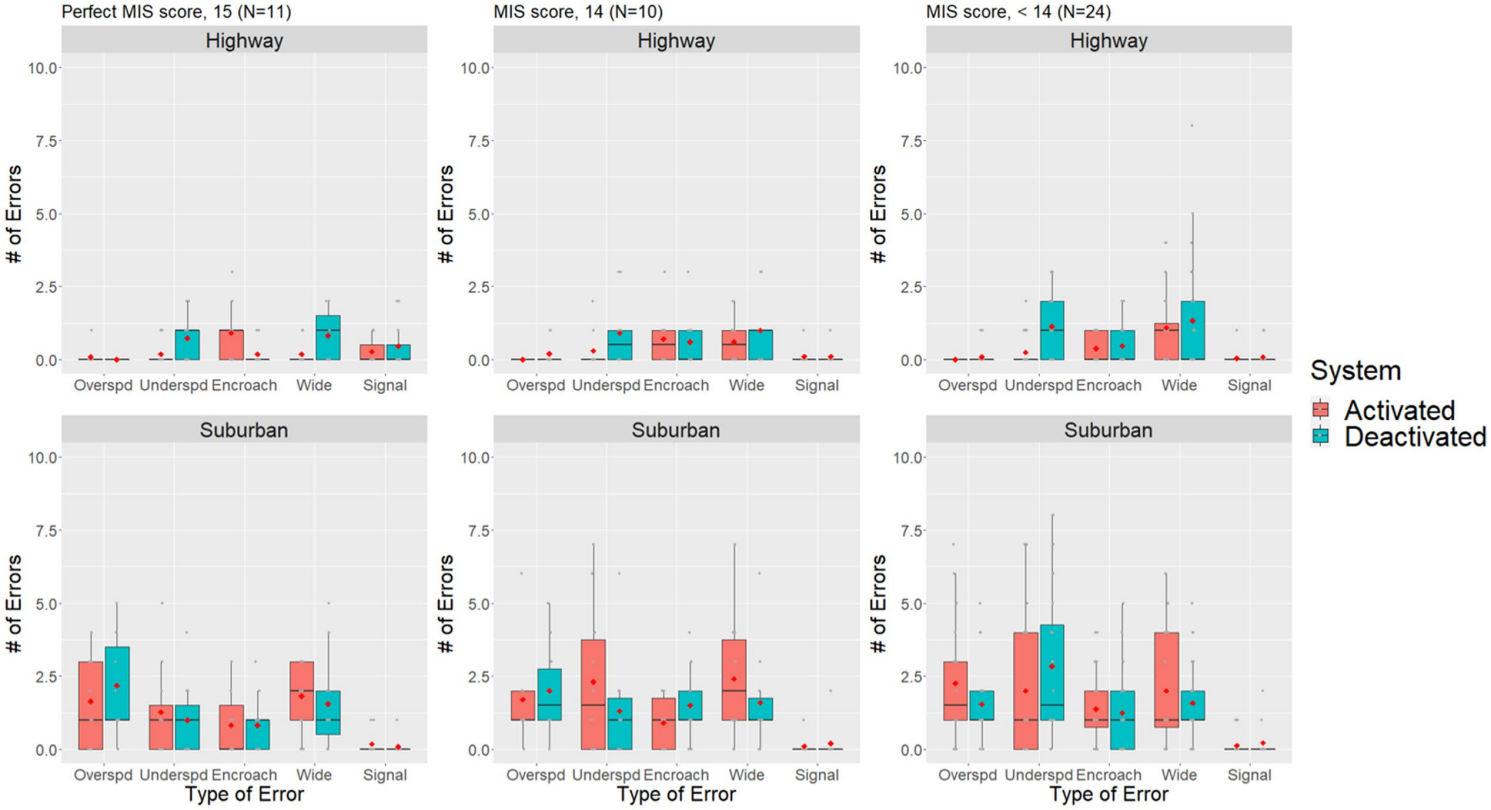
Boxplot of the number of errors for different MIS score individuals compared across driver assistance system status and road segment conditions. The red dot denotes sample means, grey dots denote individual observations.

Figure 5 shows that individuals with compromised memory skills (i.e., MIS < 14) may make slightly more driving errors overall while driving on the highway. The driver assistance systems effectively decreased the number of underspeeding errors for all groups during highway driving. Similarly, there appears to be an increasing trend of errors as MIS scores decrease during suburban drives, but the benefits of the driver assistance systems are, once again, less pronounced.

## 4. Discussion

The objective of this study was to examine the impact of IVIS and ADAS on driving errors in participants with PD, via a preliminary *descriptive analysis of on-road errors* detected by in-vehicle kinematics and the assessment data of the driver rehabilitation specialist (DRS). Specifically, we investigated an initial sample of 45 adults with diagnosed PD and compared the number of errors made during an on-road driving course.

The study reveals interesting findings in that IVIS and ADAS are more effective in reducing driving errors during the highway vs. the suburban section drives. Two important facts need to be considered when discussing the total number of driving errors: First, the ACC, only used for the highway section, might have accounted for most of the reduction in driving errors, and as such further investigation will be necessary to account for the contribution of the ACC in the overall variance of error reduction. Second, these findings are descriptive in nature and reflect an interim analysis—and as such no conclusion can yet be made about the effectiveness of the IVIS or ADAS systems for either the highway or the suburban drive. However, these findings are partly supporting our hypothesis, that is, drivers with PD will make fewer errors when ADAS is activated—as demonstrated in the highway section.

From the signaling data, we posit that the blind spot detection may not necessarily change the number of signaling errors made, in either the activated or deactivated condition of the IVIS, and this finding holds true regardless of the road section. We are not certain why this finding manifests but may pose that signaling is a proxy variable for executing divided and/or selective attention (9–12). Therefore, the cognitive load necessary to appropriately perceive, interpret, and correctly activate the turn signal—may not be offset by the visual display of the blind spot detection warning—and as such the IVIS, that only provides information to the driver, may not render the same effect as the ADAS where that system actively takes over the function of one of the primary controls (i.e., acceleration, deceleration, and lane maintenance). Furthermore, the BSM warning is located on the mirror, thus it only provides guidance to the driver when they are already checking their mirrors.

Based on the descriptive data, underspeeding errors were lower when the ACC system was active in the highway segments, while no differences were present between the system activated and deactivated conditions in the suburban drive. Whereas, in the suburban conditions, more wide lane departure errors and overspeeding errors occurred when the driver assistance system was activated than when it was deactivated. We render caution in interpreting this finding, as it is possible that automation complacency (i.e., becoming over-reliant on the automation beyond its capabilities or reliability) may have led to more lane departures, but the data are currently inadequate to justify this assertion. Moreover, it is also possible that the differences are not significant, as the SDs are currently all relatively large. But taken together, these results suggest that systems that directly takes over a portion of the driving task (e.g., ACC) can result in reductions in the corresponding driving error (i.e., under or over speeding). However, when the driving support is only a warning (e.g., LDW) or only partial support (e.g., LSA), errors may still occur.

The negative correlations between the total MoCA score and the Memory Index Score (MIS) of the MoCA with the total number of driving errors, suggest that the number of driving errors increased as cognitive ability and delayed recall ability, in particular, decreased. This is not a surprising finding, but rather a confirmation of our clinical expectations (8, 17, 36, 55, 56). Still, the extant literature on cognition and on-road driving errors has not yet documented this relationship—especially between the subcomponents of the MoCA and driving errors.

The nonsignificant correlations between the UPDRS rigidity and bradykinesia scores and total number of driving errors suggest that without driver assistance systems, cognitive ability had the largest effect on total number of driving errors, particularly memory ability as measured through the MIS. However, when these systems were activated, differences between individuals were not as impactful, suggesting that driver assistance systems may help compensate for the negative effects of individual decrements in cognitive ability. Noteworthy to mention, and consistent with the prevailing literature, we observed that all participants in the study completed the drive with their medication in an “on” state, which may explain the lack of differences due to the motor control factors (16, 21, 22). However, our preliminary results do suggest that ADAS and IVIS technologies in current on-the-market vehicles may provide positive benefits for individuals who are living with PD and are receiving treatment for their motor symptoms.

Our early reasoning pertaining to the MIS and driving error data is that these results are consistent with our earlier findings that cognitive impairment are the primary individual difference factor influencing driving errors in our sample, with memory impairment potentially having the greatest impact. Moreover, the benefits of the driving automation are the strongest when the system takes over a driving task, such as with ACC in the highway driving segments. Although too preliminary to make any inferences, this is an exciting finding. Specifically, we know that people with PD have *tactical driving skill impairments* (21), such that during a driving task, functions related to steering, braking, accelerating, stopping or controlling the vehicle, may be impaired. Specifically, drivers with PD may have speeds too high or too low when merging on a highway, or when they need to maintain a safe headway distance, or when they need to anticipate and adjust to traffic stimuli (6, 21, 23, 34, 57). Thus, given that the ACC function when activated mitigated the number of errors, particularly during the highway drive, it looks as if the ACC may hold potential benefits for the driver with PD. Particularly, the ACC *automatically adjusts the speed of the vehicle*, through deceleration or acceleration, so the driver with PD can overcome *set shifting*, *judging of gaps, memory recall* and *processing speed* demands (6, 21, 23, 34, 57), to *maintain a safe headway distance and to maintain their vehicle within the flow of traffic.* Of course, this assumption will have to be validated when the entire sample of our drivers with PD (*N* = 105) has completed their on-road exposure.

### 4.1. Limitations

Study limitations include self-selection bias, convenience sampling resulting from focusing on a major PD treatment clinic in the city, participants’ apprehension about completing on-road interventions, and demographic factors that restrict the generalizability of the findings. Furthermore, there were a number of confounding factors that may have impacted the data collection and analysis. Firstly, driving errors were identified by the onboard experimenter, who was a trained DRS. While DRS’s identify on-road driving errors as part of their role, there may be variability in what individual DRS’s include in their error counts. Second, there were natural variations in traffic density and weather conditions during the experimental drives that may have impacted the participant’s driving error. Third, this current study examined the effects of the IVIS and ADAS technologies when they were active during the experiment, but we did not examine the participant’s proficiencies in activating the systems or their ability to decide when they should engage the systems. Finally, our current analysis focused on driving errors, but did not explicitly measure the specific activity limitations related to driver tasks and how those were impacted by the ADAS and IVIS technologies. While our results provide support for the possible benefits of these technologies for individuals with PD, further work could highlight the specific driver activities (e.g., pedal control, speed judgment, etc.) that are being supported by driver assistance systems.

### 4.2. Strengths

The best evidence to ensure drivers with PD can continue to drive is sparse at best, and best practices are deficient in overcoming the PD-related functional declines necessary to drive safer and longer. Technology-based intervention to extend driver fitness, modify driver behavior (41, 43, 44), mitigate the PD-related factors affecting driving (9, 17, 22, 45), and improve community accessibility, is now a plausible reality—and this study is one of the first to examine the impact of in-vehicle technology on the driving errors of people with PD, in an on-road vehicle and in real world traffic situations. We have used a comprehensive approach, including a multi-disciplinary team (rehabilitation scientists, occupational therapists, driver rehabilitation specialist, human factors engineer, and movement disorder neurologists) to examine, understand and interpret the driving performance of people with PD via the use of in-vehicle technology. We have also utilized an assessment of on-road performance via DRS determination and associated it with individual differences in cognitive and physical ability—a comprehensive approach not often used in the literature.

## 5. Conclusion and future work

Although, we cannot make conclusive decisions for people with PD who drive with in-vehicle technology, our findings point to exciting opportunities that particularly ADAS, already available in vehicles, can be applied to help offset driving deficits, especially on the highways, for drivers with PD, while potentially also enhancing their driving safety. Our results found that speeding errors may particularly benefit from current in-vehicle technologies, but our findings are only descriptive and need to be interpreted as such. Furthermore, individual differences in cognitive ability appeared to be more impactful on driver error than differences in physical ability. However, ADAS and IVIS technologies appear to mitigate the effects of lower cognitive ability. These results lay the foundation for future research to build upon and further investigate the effectiveness of in-vehicle technologies in enhancing the driving performance of individuals with PD.

## Data availability statement

The raw data supporting the conclusions of this article will be made available by the authors, without undue reservation.

## Ethics statement

The studies involving humans were approved by University of Florida Gainesville Health Science Center Institutional Review Board (IRB-01). The studies were conducted in accordance with the local legislation and institutional requirements. The participants provided their written informed consent to participate in this study.

## Author contributions

WG: conceptualization, methodology, data collection, investigation, writing–draft preparation, and writing–reviewing and editing. HZ: data collection, data processing, data visualization, writing–draft preparation, and writing–reviewing and editing. BG: data collection and writing–reviewing and editing. BP: participant recruitment, data collection, and writing–reviewing and editing. AR-Z: participant recruitment, data collection, investigation, and writing–reviewing and editing. SW: data collection, writing–draft preparation, and supervision. MJ: participant recruitment and enrollment, data collection, and writing–reviewing and editing. YL: data collection, participant recruitment and enrollment, and writing–draft preparation. SC: conceptualization, methodology, participant recruitment, writing–draft preparation, writing–reviewing and editing, and supervision. All authors contributed to the article and approved the submitted version.

## Supplementary material

Supplementary Table 1 Error logging form used by Driver Rehabilitation Specialist during on-road experiment.

Supplementary Figure 1 Flowchart of the experimental procedure.
