# Supplementary material for: Does in-vehicle automation help individuals with Parkinson’s disease? A preliminary analysis
Source: Front Neurol. 2023 Oct 13;14:1225751. doi: 10.3389/fneur.2023.1225751 (PMC10603248; doi:10.3389/fneur.2023.1225751)
Supplement: Supplementary Table 1 — Error logging form used by Driver Rehabilitation Specialist during on-road experiment. [file Data_Sheet_1.PDF]

NIDILRR/PD Study Error Sheet

Participant #:

Date and time of drive:____

Zone	Directions & Roadways	Length (miles)	Speed		Lane Maintenance		Signaling	Comments
			Over	Under	Encroach	Wide		
Orientation to vehicle	UF Fixel parking lot	0.60						
	RIGHT on SW Williston Rd							
Suburban roadways	SW Williston Rd → S Main St (Straight)	2.35						
	LEFT on S Main St							
	S Main St → SW 16th Ave (Straight)	1.22						
	LEFT on SW 16th Ave							
	SW 16th Ave → SW 13th St (Straight)	0.72						
	LEFT on SW 13th St							
	SW 13th St → SW Williston Rd (Straight)	1.50						
	RIGHT on SW Williston Rd							
	SW Williston Rd → I75 North entrance (Straight)	2.14						
	RIGHT onto I75 North on-ramp							
Highway roadways	I-75 North → W Newberry Rd exit	4.82						
	LEFT on W Newberry Rd							
Suburban roadways	W Newberry Rd → Hardee's parking lot (Straight)	0.14						
	RIGHT into Hardee's parking lot							
	Hardee's parking lot → W Newberry Rd	0.05						
	Exit straight out of Hardee's parking lot onto I75 South entrance							
Highway roadways	I-75 South → SW Willison Rd	4.93						
	LEFT on SW Williston Rd							

NIDILRR/PD Study Error Sheet Participant #:

Date and time of drive:_______________________________________

Zone	Directions & Roadways	Length (miles)	Speed		Lane Maintenance		Signaling	Comments
			Over	Under	Encroach	Wide		
Suburban roadways	SW Williston Rd → S Main St (Straight)	2.94						
	LEFT on S Main St							
Suburban roadways	S Main St → SW 16th Ave (Straight)	1.22						
	LEFT on SW 16th Ave							
	SW 16th Ave → SW 13th St (Straight)	0.72						
	LEFT on SW 13th St							
	SW 13th St → SW Williston Rd (Straight)	1.50						
	RIGHT on SW Williston Rd							
	SW Williston Rd → UF Fixel Institute (Straight)	1.71						
	LEFT into UF Fixel Institute							

Driving error calculations:

Speeding	
• Over	
• Under	
Lane Maintenance	
• Encroach	
• Wide	
Signaling	
**Total Number of Errors**	

Notes:

Weather:

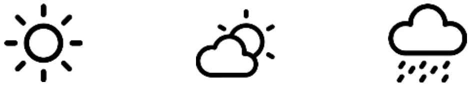

Experience with (comment):
- IVIS only:
- ADAS only:
- IVIS and ADAS concurrently:

Drive 1: IVIS/ADAS - ON OFF

Drive 2: IVIS/ADAS - ON OFF

Revised 8April2021
