# Supplementary figures and images for: Does in-vehicle automation help individuals with Parkinson’s disease? A preliminary analysis
Source: Front Neurol. 2023 Oct 13;14:1225751. doi: 10.3389/fneur.2023.1225751 (PMC10603248; doi:10.3389/fneur.2023.1225751)

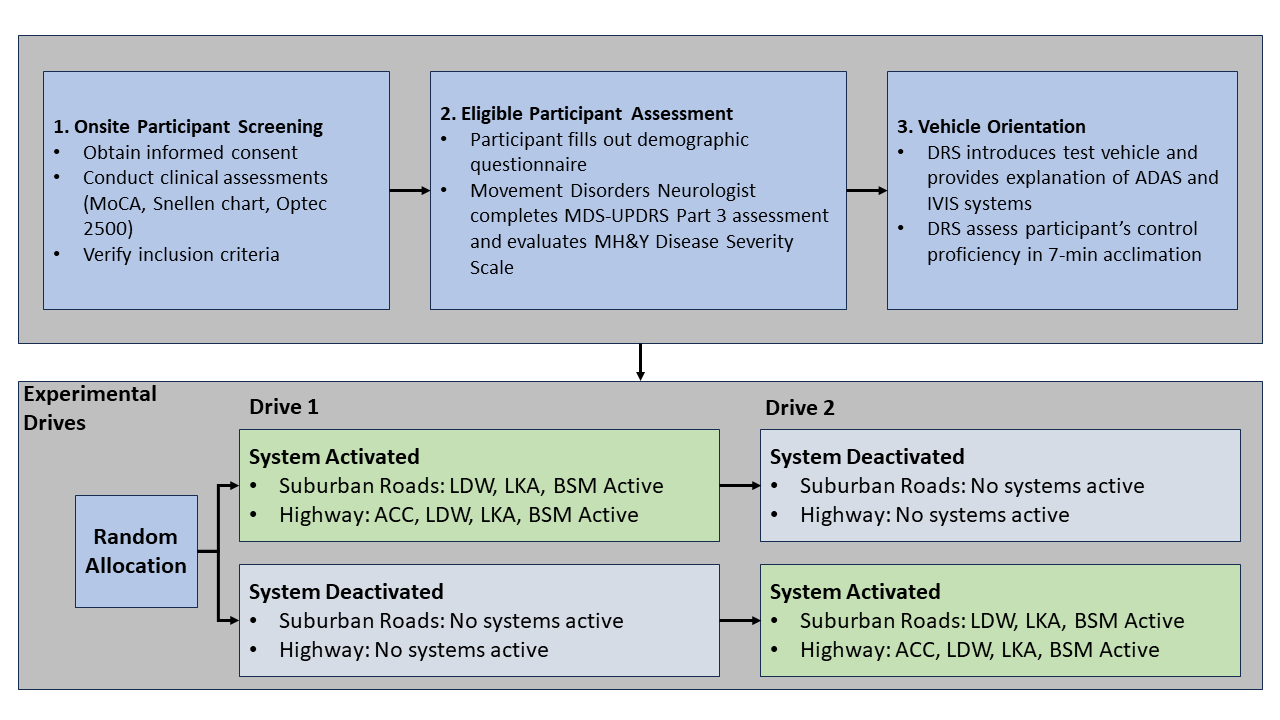

Supplement: Supplementary Figure 1 — Flowchart of the experimental procedure. [file Supplementary_Figure.TIF]
